# A new era of macrophage-based cell therapy

**DOI:** 10.1038/s12276-023-01068-z

**Published:** 2023-09-01

**Authors:** Yi Rang Na, Sang Wha Kim, Seung Hyeok Seok

**Affiliations:** 1 Translational Immunology Laboratory, Department of Transdisciplinary Medicine, Seoul National University Hospital, Seoul, South Korea; 2 Macrophage Laboratory, Department of Microbiology and Immunology, and Institute of Endemic Disease, Seoul National University College of Medicine, Seoul, 110-799 South Korea; 3 Department of Biomedical Sciences and Seoul National University College of Medicine, Seoul, Republic of Korea; 4 Cancer Research Institute, Seoul National University College of Medicine, Seoul, South Korea

**Keywords:** Drug development, Stem-cell therapies

## Abstract

Macrophages are essential innate immune cells found throughout the body that have protective and pathogenic functions in many diseases. When activated, macrophages can mediate the phagocytosis of dangerous cells or materials and participate in effective tissue regeneration by providing growth factors and anti-inflammatory molecules. Ex vivo-generated macrophages have thus been used in clinical trials as cell-based therapies, and based on their intrinsic characteristics, they outperformed stem cells within specific target diseases. In addition to the old methods of generating naïve or M2 primed macrophages, the recently developed chimeric antigen receptor-macrophages revealed the potential of genetically engineered macrophages for cell therapy. Here, we review the current developmental status of macrophage-based cell therapy. The findings of important clinical and preclinical trials are updated, and patent status is investigated. Additionally, we discuss the limitations and future directions of macrophage-based cell therapy, which will help broaden the potential utility and clinical applications of macrophages.

## Cell therapy: the potential of macrophages, innate immune system cells

Treating disease with the white blood cells called macrophages, in some cases genetically modified, could become an effective clinical option for a range of diseases. Macrophages are cells of the innate (non-specific) immune system that can destroy diseased cells or damaging materials. They can also enhance tissue repair by releasing growth factors and anti-inflammatory molecules. Seung Hyeok Seok and colleagues at Seoul National University in South Korea review the laboratory research and clinical trials that are investigating the potential of macrophages in cell-based therapies. In some trials, macrophages have outperformed cell therapies using the more widely hailed stem cells. Conditions targeted include kidney disease, stroke, arterial disease and cancer. Genetic modification can focus macrophage activity on specific conditions. The authors also survey the wide range of patent activity involving macrophage therapy.

## Introduction

### History of cell-based therapeutics

Deploying working resources is an exciting and necessary solution when further improvements are not expected from existing pools. Cell therapy conceptually meets this expectation by delivering ‘workable’ cells within the body as medical treatments. The first cell therapy in modern medical history was the intravenous transfusion of whole blood from a donor to a recipient in 1900^1^. Based on the identification of human blood groups, allogeneic blood transfusion became a consolidated medical practice during the First World War and remains a central component of medicine today. Bone marrow transplantation (BMT) facilitated the era of stem cell therapy via the action of long-term self-renewing hematopoietic stem cells transferred from donor to patient^2^. For decades, cell therapy was predominantly limited to BMT for hematological diseases and epidermis transplantation for large burns^3^. Recently, however, cell-based therapeutics have experienced exponential growth within the pharmaceutical industry^4^. A noteworthy clinical application of genetically engineered cells, chimeric antigen receptor T cell (CAR-T) therapy, has emerged and will be an important component of cell therapy in the future^5^.

### Current categories of cell therapy in clinics

With regard to cell origin, the donor and the recipient can be the same (autologous transplantation) or different individuals (allogeneic transplantation)^3^. Although they do not require cell transplantation, the mobilization of autologous cells is also considered cell therapy. Regarding the differentiation status of cellular sources, stem cells and fully differentiated cells are both applied.

Stem cells are mainly obtained from samples of tissues (skin, cornea, adipose tissue) obtained through biopsy and blood or cord blood samples^6–8^. Embryonic stem cells and induced pluripotent stem cells (iPSCs) will be used more extensively in the future if their utility is proven to outweigh the ethical problems^9^. Except for blood, bone marrow and adipose tissue-derived stem cells, most patients require ex vivo expansion of cells to obtain appropriate therapeutic efficacy^10^. For example, in 2014, the first cell therapy product to receive market authorization in Europe was that for cornea transplantation, which requires two interventions: (1) limbal biopsy and (2) in vitro cell expansion to generate the new cornea^11^.

Directly applying differentiated cells is considered the halfway point between cell and organ transplantation. Islet transplantation is an established therapy for patients with diabetes and has shown limited long-term success rates^12^. Several clinical trials using differentiated cells have been conducted in patients with heart infarction (cardiomyocytes)^13^, Parkinson’s disease (dopaminergic neurons)^14^, and Duchenne muscular dystrophy (myoblasts)^15^. These trials had inadequate clinical outcomes, and overcoming the barrier of cellular sources remains problematic. Obtaining differentiated cells from genetically modified iPSCs is an intriguing solution but currently requires further development.

CAR-T therapy uses engineered DNA constructs introduced into patient T cells to redirect their cytotoxicity to tumor cells that bear CD19, a B lymphocyte-associated antigen^16^. This therapy has led to significant advancements in the use of differentiated cells. Engineered alterations in cellular function potentiated the therapeutic use of specific cell types^4^. Innovations in engineering disciplines are currently being explored, and some of these approaches have been successfully used to generate commercialized products^17^, although many remain at a preclinical stage.

### Macrophage-based cell therapy

Immune cells have specialized characteristics. Their unique ability to move throughout the body enables them to actively search for their target sites and perform their specific roles in the body; this is what we aim for in immune cell-based therapeutics. In this regard, macrophages have great potential as a cell source in cell therapy.

Macrophages are strategically distributed throughout the body as tissue-resident innate immune cells. They perform a vital homeostatic role as prodigious phagocytic cells that clear intruding pathogens and large amounts of endogenous harmful materials, such as apoptotic cells, dying erythrocytes, amyloid beta and surfactants, to maintain normal organ function^18^. Macrophages are highly heterogeneous cells that can rapidly change their function in response to local microenvironmental signals^19^. They have an extremely plastic nature in vivo and are involved in many human diseases with both protective and pathogenic functions^19^. Insights into the development of macrophage-based cell therapies have focused on their notable actions, such as promoting tissue regeneration and clearing cancer cells or pathogens^20^.

In this review, we update and discuss the current developmental status of macrophage-based cell therapy. In line with the characteristics of macrophages, a wide variety of target diseases are briefly introduced. We also discuss their limitations along with potential future directions.

### Clinical trials on macrophage-based therapy

A clinical trial search in ClinicalTrials.gov was conducted with one keyword (Macrophage). In total, 366 search results were found, and it was manually determined whether the trial was relevant to macrophage cell therapy. Only 11 out of the 366 search results were macrophage cell therapy-related clinical trials, and these 11 trials were included in this review (Table 1). Among the 11 selected clinical trials, nine (81.8%) were regarding the adoptive transfer of macrophages and ex vivo polarization with different strategies. Two studies (18.1%) were chimeric antigen receptor-macrophage (CAR-M) trials. Not all clinical trials reported the patient response publicly, either through ClinicalTrials.gov or a paper publication. Interestingly, all studies with publicly reported response data focused on ‘ex vivo polarization and adoptive transfer’.

**Table 1:** Clinical trials of macrophage-based cell therapy.

Classification	Registration number	Title	Phase	Target disease	Treatment approach	No. of patients	Responses	Adverse effects
Ex vivo polarization and adoptive transfer	NCT00507364	Treatment of anal fissure by activated human macrophages	Phase 3	Chronic anal fissure	Macrophage suspension (0.05 mL/injection) injected at 0.5–1 cm from the edge of the ulcer	199	27% and 6% of the macrophage-treated and control groups, respectively, were completely healed	No adverse effects were noted
	NCT01845350	Safety of autologous M2 macrophages in the treatment of nonacute stroke patients	Phase 1	Ischemic stroke; hemorrhagic stroke	Generation of autologous M2 macrophages from peripheral blood of nonacute stroke patients and intrathecal introduction of the autologous M2 macrophages	13	75% and 18% of the group receiving M2 macrophages and the control group showed obvious and positive improvement, respectively	No serious adverse effects were noted
	NCT02085629	The ONE Study M Reg Trial	Phase 1, Phase 2	Renal failure (end stage)	M registered (monocyte-derived regulatory macrophages) from donor infused to recipients	8	Regulatory cell therapy is associated with fewer infectious complications but showed similar rejection rates in the first year	No safety concerns when compared with the reference group trial
	NCT00468000	Use of Ixmyelocel-T (formerly vascular repair cells [VRC]) in patients with peripheral arterial disease to treat critical limb ischemia	Phase 2	Peripheral arterial disease	Adoptive transfer of Ixmyelocel-T including autologous bone marrow-derived activated macrophages	86	Major amputation occurred in 19% of tissue repair cell-treated patients compared to 43% in controls	No difference in adverse events between the groups
	NCT00505219	Ixmyelocel-T treatment of patients with osteonecrosis of the femoral head	Phase 3	Osteonecrosis		11	Not reported	Not reported
	NCT01483898	An efficacy and safety study of ixmyelocel-T in patients with critical limb ischemia (cli)	Phase 3	Critical limb ischemia		41	Not reported	Not reported
	NCT01670981	An efficacy, safety and tolerability study of ixmyelocel-T administered via transendocardial catheter-based injections to subjects with heart failure due to ischemic dilated cardiomyopathy (IDCM)	Phase 2	IDCM		114	The transendocardial delivery of ixmyelocel-T in patients with heart failure and reduced ejection fraction due to IDCM resulted in a significant reduction in adjudicated clinical cardiac events compared with placebo, leading to improved patient outcomes	Adverse effects were markedly decreased in the treatment group
	NCT01020968	Use of Ixmyelocel-T (Formerly Catheter-based Cardiac Repair Cell [CRC]) treatment in patients with heart failure due to dilated cardiomyopathy	Phase 2	DCM		22	Intramyocardial injection with ixmyelocel-T reduces major adverse cardiovascular events and improves symptoms in patients with IDCM but not in patients with nonischemic DCM	Adverse effects were markedly decreased in the treatment group
	NCT00765518	Use of ixmyelocel-T (formerly cardiac repair cell [CRC] treatment) in patients with heart failure due to dilated cardiomyopathy (IMPACT-DCM)	Phase 2	DCM		39		
CAR-M	NCT04660929	CAR-macrophages for the treatment of HER2-overexpressing solid tumors	Phase 1	HER2-overexpressing solid tumors	Anti-HER2 CAR macrophage (CT-0508) treatment in combination with pembrolizumab	48	-	-
	NCT03608618	Intraperitoneal MCY-M11 (mesothelin-targeting CAR) for the treatment of advanced ovarian cancer and peritoneal mesothelioma	Phase 1	Relapsed/refractory ovarian cancer, Peritoneal mesothelioma, etc.	Intraperitoneal infusion of MYC-M11 (mesothelin-targeting CAR)	14	Not reported	Not reported

*DCM* dilated cardiomyopathy, *IDCM* ischemic dilated cardiomyopathy.

Most of the clinical trials that used ex vivo polarization and adoptive transfer of macrophages are in phase 2 or 3, and the target diseases are well known, including cardiomyopathy, osteonecrosis, limb ischemia, stroke, arterial disease, and chronic anal fissure, indicating the clinical usefulness of the methodology in regenerative medicine. One of the representative studies was entitled “Treatment of Anal Fissure by Activated Human Macrophages” (registration number: NCT00507364) and was a phase 3 trial consisting of chronic anal fissure treatment^21^. In this trial, 199 patients were divided into control and macrophage-treated groups. In the macrophage-treated group, a macrophage suspension (0.05 mL/injection) was injected 0.5–1 cm from the edge of the ulcer. Complete recovery was achieved in 27% of the macrophage-treated group, whereas only 6% of the control group showed complete recovery. No adverse effects were noted in the trial. Another trial with publicly reported response data was a phase 1 trial entitled “Safety of Autologous M2 Macrophages in the Treatment of Non-Acute Stroke Patients” (registration number: NCT01845350), which focused on ischemic and hemorrhagic stroke treatment^22^. Autologous peripheral blood mononuclear cells were obtained from patients with nonacute stroke, polarized to M2 macrophages, and injected intrathecally by a lumbar puncture after premedication with dexasone. Thirteen patients were enrolled and divided into control and treatment groups. Clear improvement in the NIH Stroke Scale/Score (NIHSS) was observed in 75% of the treatment group and 18% of the control group. No adverse effects related to cell therapy were noted throughout the trial. In the phase 2 trial “Use of Ixmyelocel-T (Formerly Catheter-based Cardiac Repair Cell [CRC]) Treatment in Patients with Heart Failure Due to Dilated Cardiomyopathy”, Ixmyelocel-T, including autologous bone marrow-derived activated macrophages, was administered to patients through intramyocardial injection^23^. Positive effects with improved symptoms were found in patients with ischemic dilated cardiomyopathy but were not found in the nonischemic population. Major adverse cardiovascular events were markedly decreased in the treatment group of ischemic patients, indicating good efficacy of Ixmyelocel-T.

There were no publicly reported response data from any of the studies on CAR-M therapy; it is necessary to note that clinical trials on the technology are in the early stages of development. One representative clinical trial is “CAR-Macrophages for the Treatment of HER2-Overexpressing Solid Tumors”, which is an ongoing phase 1 trial targeting HER2-overexpressing solid tumors. In this clinical trial, CAR-M targeting HER2 was constructed (CT-0508) and intravenously injected into the treatment group. As it is an ongoing trial and the recruitment status is “recruiting”, no response results could be expected as yet. Another important phase 1 clinical trial, “Intraperitoneal MCY-M11 (Mesothelin-targeting CAR) for the Treatment of Advanced Ovarian Cancer and Peritoneal Mesothelioma”, targeted relapsed/refractory ovarian cancers, including peritoneal mesothelioma, fallopian tube adenocarcinoma, adenocarcinoma of the ovary, and primary peritoneal carcinoma. MYC-M11 (mesothelin-targeting CAR) was administered by intraperitoneal infusion to 14 participants; the response data have yet to be publicly reported through ClinicalTrials.gov or a publication of the study findings.

Another important clinical trial should be noted; however, it was conducted in the UK and is not presented in Table 1. Research groups at the University of Edinburgh successfully completed autologous macrophage therapy for liver cirrhosis^24^. They conducted a phase 1 dose escalation trial of autologous macrophage therapy in nine adults and found that all participants survived and were transplant-free at the one-year follow-up.

### Patent status

To identify the international patent status of macrophage-based cell therapy, we conducted a patent analysis and an analysis of market and technology trends. MJL Bio Co. retrieved patent samples with a priority date before 15 June 2021 using a series of search terms, including macrophage, treat, prevent, wound, regeneration, recover, repair, anticancer and inhibit or suppress. In total, 9045 patent documents were initially obtained. From these, we excluded 8833 patents. These were patents for which the aim was the modulation of in vivo resident macrophages by drugs or where macrophages were not used for cell therapy. Ultimately, 212 patent documents were included for further interpretation (Supplementary Table 1).

**Fig. 1:**
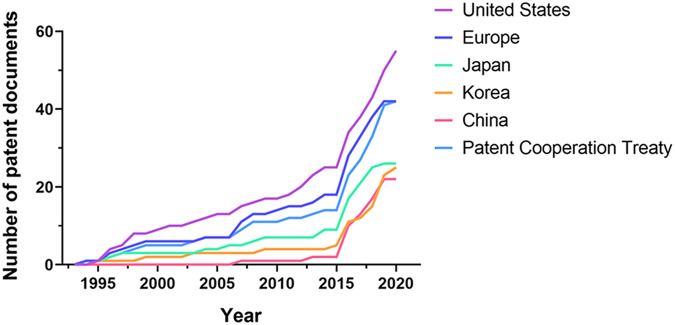
Publication trend of macrophage-based cell therapy patents. Macrophage cell therapy patent documents by publication year and the top six countries in which assignees applied.

Looking at the overall application trend by year in the field of macrophage cell therapy, research has been conducted since 2015 (Fig. 1). Recently, considering the existence of undisclosed cases, macrophage cell therapy is on the rise and in the early stages of technological growth worldwide. The top inventors are located in the United States (26%), Europe (20%) and PCT (20%). Among them, the University of Pennsylvania developed CAR-M for the treatment of cancer (Table 2). Duke University has the second-highest number of inventions and primarily focuses on methods for treating cancers and pathogen infections using antigen-presenting cells loaded with RNA. Most patents were related to cancer, but many were related to regenerative medicine. For example, XCELL medical solutions and INSERM applied for patents with regenerative diseases as an indication. XCELL’s patent provides in vitro methods to induce macrophage polarization in an M2 phenotype that overexpresses NGAL and IL-10, which is useful for tissue recovery.

**Table 2:** List of notable patents on macrophage-based cell therapy.

No	Inventor	Patent code	Publication year	Title	Target disease	Modification
1	INSERM (Institut National De La Sante Et De La Recherche Medicale) | Centre National De La Recherche Scientifizue (CNRS)	WO2008-084069	2008	A Method for Expanding Monocytes	Monocyte expansion for a disease resulting from a deficiency in the monocyte compartment (cancer, acute or acquired immuno-deficiencies, chronic or acute injury, wounds, degenerative diseases, autoimmune diseases, chronic inflammatory diseases, atherosclerosis, poly- and osteoarthritis, osteoporosis, infectious diseases (e.g., viral or bacterial infections), and metabolic diseases)	Not modified
2	Xcell Medical Solutions, S.L.	WO2018-055153	2017	Cell Therapy with Polarized Macrophages for Tissue Regeneration	Use of polarized macrophages for tissue regeneration	Not modified
3	The Trustees of the University of Pennsylvania	WO2017-019848	2020	Modified Monocytes/Macrophage Expressing Chimeric Antigen Receptors and Uses Thereof	Cancer (solid tumor or hematologic malignancy)	Modified (CAR)
4	Duke University	WO1997-041210	1997	Methods for Treating Cancers and Pathogen Infections Using Antigen-Presenting Cells Loaded with RNA	Cancer or infectious diseases	Modified
5	LFB Biotechnologies | I.D.M. Immuno-Designed Molecules	WO2008-146165	2008	Kit of Parts for the Treatment of Cancer or Infectious Diseases	Cancer or infectious diseases	Not modified
6	Vlaams Interuniversitair Instituut Voor Biotechnologie VZW	WO1999-021968	1998	Inducible Cellular Immunity Through Activation of Th1 and Suppression of Th2 Responses by Macrophages	Macrophage usage for cellular immune response modulation (Th1 activation and/or Th2 suppression)	Not modified
7	The Arizona Board of Regents on Behalf of the University of Arizona	US (14/213780)	2014	Methods and Compositions for Treating Congestive Heart Failure	Congestive heart failure	Not modified
8	Keele University	WO2007-113572	2007	Targeted Therapy	Targeted therapy for diseased tissue using monocytes or macrophages with magnetic material	Not modified
9	The University Court of the University of Edinburgh	WO2018-051136	2017	Macrophage-Based Therapy for Use in the Treatment of Liver Injury	Liver injury	Not modified
10	Wisconsin Alumni Research Foundation	WO2018-183825	2018	Generation of Therapeutic Cells Using Extracellular Components of Target Organs	Ex vivo generation and use of tissue-specific anti-inflammatory macrophages	Not modified
11	Verseau Therapeutics, INC.	WO2020-006385	2019	Composition and Method for Controlling the Inflammatory Phenotype of Monocytes and Macrophages, and Immunotherapy Use Thereof	Phenotype modulation and immunotherapeutic use of macrophages and monocytes	Not modified
12	Fropharm GMBH	WO2017-153607	2017	New Immunoregulatory Cells and Methods for Their Production	Treatment of different immunological and nonimmunological diseases and conditions	Not modified
13	Trizell GMBH	WO2019-053091	2017	Regulatory Macrophages for the Treatment of Angiopathies	Regulatory macrophages for angiopathy treatment	Not modified
14	Ruprecht-Karls-Univeristät Heidelberg | Universität ULM	WO2012-062930	2010	Use of Interleukin 10 mRNA Transfected Macrophages in Anti-Inflammatory Therapies	Anti-inflammatory therapy	Modified (others)
15	Cellular Approaches, INC	WO2019-055697	2018	Autologous and Allogenic Macrophages and Monocytes for Use in Therapeutic Methods	Complicated intra-abdominal infection	Not modified

Regarding genetic modification status, 107 patents (55%) were for unmodified macrophages, and 83 (43%) were for modified macrophages. Unmodified macrophages were most frequently applied as activated macrophages and M2 macrophage types. Modified macrophage-related patents were mainly aimed at CAR-M therapy, with 25 studies.

### Animal experimental trials

The most widespread example of the experimental success of macrophage cell therapy is in the pulmonary alveolar proteinosis (PAP) disease model^25^. This disease is prevalent in children who have mutations in *CSF2RA* or *CSF2RB*, encoding GM-CSF receptor α or β, respectively, which cause hPAP by impairing GM-CSF-dependent surfactant clearance by alveolar macrophages, resulting in progressive surfactant accumulation in alveoli and hypoxemic respiratory failure^26,27^. There are currently no pharmacological therapies for hPAP, and surfactants must be removed by whole-lung lavage, an inefficient and invasive procedure to physically remove excess surfactant. Suzuki et al. showed the efficient therapeutic potential of pulmonary macrophage transplantation (PMT) of either wild-type or *Csf2rβ* gene-corrected macrophages without myeloablation in *Csf2rβ* mutant mice. PMT was safe and efficient, and only one administration was required to treat lung disease. These exciting results highlighted the need for the development of allogeneic macrophage-based cell therapies based on genetic modifications.

We previously showed the obvious regenerative capacity of wild-type macrophages when transferred into *Ptger4* mutant mice with a defect in proper intestinal epithelial regeneration postinflammation^28^. This study thereby highlighted the need for allogeneic sources of macrophage cell therapy in patients with IBDs who carry genetic mutations at the *PTGER4* locus.

Neurodegenerative diseases are another promising target for macrophage cell therapy because stem cell engraftment has clear limitations due to the large size of the human brain; moreover, the need for stereotactic injection complicates the homogeneous distribution of grafted cells even when highly migratory cells such as oligodendrocyte precursors are transplanted^29^. Transferred bone marrow cell-derived macrophages showed the capacity to efficiently distribute to the brain and clear accumulated glucosylsphingosine in place of malfunctioning microglia in a Parkinson’s disease model^30^.

**Fig. 2:**
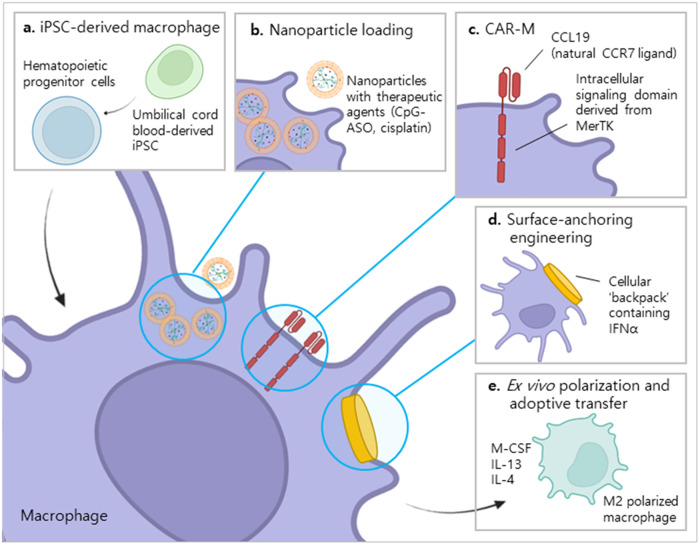
Promising in vivo studies of macrophage-based cell therapy. Various attempts to increase the efficiency of macrophage-based cell therapy have been reported. Promising in vivo studies in the previous 5 years (2019–2023) are classified into five different groups. **a** Direct differentiation of macrophages from iPSCs enables bulk production of macrophages for therapeutic use^32^. **b** Utilizing the function of macrophages as efficient transporters, various nanoparticles with therapeutic agents can be loaded onto macrophages and delivered to target lesions^33^. **c** Diverse trials changing the target molecules and intracellular signaling domains of the existing CAR-M structure can increase the therapeutic efficacy^31^. **d** Macrophages can also be used as delivery cells by anchoring certain materials on the surface of the cell^34^. **e** Patient macrophages can be collected and artificially polarized ex vivo for successive adoptive transfer back to the patient^35,41^. iPSC induced pluripotent stem cell, CpG-ASO unmethylated cytosine-phosphate-guanine motif and anti-P-glycoprotein antisense oligonucleotide, CAR-M chimeric antigen receptor macrophage, MerTK Mer tyrosine kinase, IFN-α interferon alpha, M-CSF macrophage colony-stimulating factor.

As in vivo studies have been continuously reported in recent years, articles published from 2019 to 2023 were searched using PubMed (https://pubmed.ncbi.nlm.nih.gov/) with the search string ((macrophage[Title/Abstract]) AND (cell therapy[Title/Abstract])). In total, 263 search results were manually determined to be relevant to macrophage cell therapy. With regard to review papers, references were also considered in our search and were reviewed to determine their relevance. Most studies were related to CAR-T therapy using granulocyte-macrophage colony-stimulating factor (GM-CSF) or analyzed the effects of stem cell therapy on macrophages and were excluded from our candidate study list. Studies that included only in vitro experiments were also excluded to limit this review to studies demonstrating clinical possibilities. As a result, 38 papers were included as macrophage cell therapy-related studies and were further classified into five different groups according to methodology as follows: CAR-M, induced pluripotent stem cell (iPSC)-derived macrophages, macrophages loaded with nanoparticles; ex vivo polarization and/or adoptive transfer of macrophages, and surface-anchoring engineering of macrophages (Supplementary Table 2). Among the 38 studies, 12 were considered promising and representative and thereby selected and presented in Table 3 and Fig. 2.

**Table 3:** Promising in vivo studies of macrophage cell therapy [Published in 2019–2023].

Classification	Method	Macrophage origin	Target disease	In vivo effects
CAR-M	Anti-CCR7 CAR with cytosolic domain from Mer receptor tyrosine kinase (MerTK)	RAW264.7 cell line	4T1 breast cancer	Suppressed tumor growth and prolonged survival prolongation by preventing metastasis and inducing systemic antitumor immunity^31^
iPSC-derived macrophages	iPSC-derived macrophages polarized to M1 and M2 subtype	Umbilical cord blood CD34^+^ cells derived from iPSCs	Liver fibrosis (CCl_4_ administered mouse)	Reduced fibrosis and inflammation in liver fibrosis^32^
Nanoparticle loading	M1 polarized macrophages loaded with nanospheres composed of functional nucleic acid therapeutic (CpG-ASO) and the chemotherapeutic drug cisplatin (Pt)	RAW264.7 cell line	A549 human alveolar basal epithelial cell adenocarcinoma	Nanosphere delivery and synergistic immunostimulation by macrophages resulted in both significant antitumor performance and well-tolerated biosafety^33^
Surface-anchoring engineered	Macrophages with soft particles called ‘backpacks’ containing IFNα on the cell surface	Bone marrow-derived macrophages from BALB/c mice	4T1 breast cancer	Significantly reduced tumor growth and improved survival without significant toxicity^34^
Ex vivo polarization and adoptive transfer	Adoptive transfer of ex vivo M2 polarized peritoneal macrophages using IL-4 and IL-13	Peritoneal macrophages from C57BL/6 mice	Acute kidney injury	Ameliorated acute kidney injury by decreasing inflammatory response and promoting proximal tubular epithelial cell proliferation^55^
	Adoptive transfer of ex vivo M2 polarized bone marrow-derived macrophages using IL-4 and M-CSF	Bone marrow-derived macrophages from C57BL/6 mice	Experimental autoimmune encephalomyelitis (EAE)	Successfully relieved EAE symptoms^35^
	Adoptive transfer of ex vivo activated peripheral blood mononuclear cell-derived macrophages using mesenchymal stem cell supernatant	Peripheral blood mononuclear cell-derived macrophages from BALB/c mice	Full-thickness cutaneous wound	Significantly increased wound healing rates with less scarring^36^
	Adoptive transfer of ex vivo M1 polarized bone marrow-derived macrophages using LPS and IFN-r	Bone marrow-derived macrophages from BALB/c mice	Hepatic fibrosis caused by cystic echinococcosis (*Echinococcus granulosus* infection)	Alleviated liver fibrosis caused by persistent *Echinococcus granulosus* infection^37^
	Adoptive transfer of bone marrow mononuclear cells	Bone marrow-derived mononuclear cells from C57BL/6 mice	Heart tissue damage	Induction of regional accumulation of CCR2^+^ and CX3CR1^+^ macrophages and subsequent functional rejuvenation at the site of injured heart tissue^38^
	Adoptive transfer of ex vivo M2 polarized macrophages using extracellular vesicles from mesenchymal stromal/stem cells	Peripheral blood mononuclear cell-derived macrophages from healthy human donor	Achilles tendon rupture	Significantly promoted healing response^39^
	Adoptive transfer of ex vivo M2 polarized bone marrow-derived macrophages using tauroursodeoxycholic acid (TUDCA)	Bone marrow-derived macrophages from rats	Spinal cord injury	Significantly increased anti-neuroinflammatory effect and motor function recovery^40^
	Adoptive transfer of M2 polarized bone marrow-derived macrophages using M-CSF and IL-4, IL-10, or TGF-β1	Bone marrow-derived mononuclear cells from C57BL/6 mice	Myocardial infarction	Increased therapeutic efficacy^41^

Niu et al. (2021) generated CAR-M targeting CCR7 with a cytosolic domain from Mer receptor tyrosine kinase for tumor growth suppression, metastasis prevention, and subsequent prolongation of survival^31^. The application of anti-CCR7 CAR-M successfully resulted in the suppression of immunosuppressive cell migration from tumor tissue to distal immune organs, showing effective systemic antitumor immunity in vivo.

One issue that requires elucidation in order to use macrophages for therapeutic purposes is how to sufficiently increase the supply quantity. Since the replication capability of macrophages is limited, Pouyanfard et al. (2021) performed a new trial by directly differentiating iPSCs into macrophages to meet the needs on a large scale while still maintaining a homogenous population^32^. This method effectively induced both M1 and M2 macrophages; in particular, the resulting M2 macrophages were found to reduce fibrogenic gene expression and associated histological markers in liver fibrosis in vivo.

Because of the good penetration efficacy of macrophages into lesions, they can also be used as good transporters of loaded nanoparticles. Wang et al. (2022) used a RAW264.7 cell line polarized to M1 macrophages and loaded with nanospheres composed of the nucleic acid therapeutic and chemotherapeutic drug cisplatin. The loaded cells were injected and targeted A549 human alveolar basal epithelial cell adenocarcinoma; significant antitumor performance without significant adverse effects was observed^33^.

Shields et al. (2020) reported one of the most innovative approaches to maintaining macrophage subtypes for a longer duration based on the geometric principles of macrophage phagocytosis^34^. They anchored soft particles called ‘backpacks’ on the surface of the macrophages, which are phagocytosis-resistant owing to their morphology and, at the same time, contain IFNα to help macrophages maintain the M1 subtype for longer. The cells with ‘backpacks’ significantly reduced tumor growth and improved survival without significant toxicity when used in a 4T1 breast cancer cell model in vivo.

Most studies using ex vivo polarization and adoptive transfer generally make use of M2-polarized macrophages, which reduce the inflammatory response and simultaneously increase wound healing. Treatment using this method has been studied in a wide variety of target diseases, including acute kidney injury, autoimmune encephalomyelitis, full-thickness cutaneous wounds, hepatic fibrosis caused by cystic echinococcosis, heart tissue damage, Achilles tendon rupture, spinal cord injury, and myocardial infarction^35–41^.

### Limitations

For macrophage-based cell therapy to be successful, preclinical study and clinical trial pathways must be carefully navigated, and scientific and manufacturing hurdles must be overcome.

Two of the most common indications are cancer and regenerative diseases, indicating that the most significant characteristic of macrophages is their plasticity. They have the ability to alter their phenotypes in response to their surroundings^42^; however, because of this ability, they have an important inherent drawback. We expect CAR-M to have phagocytic ability against cancer cells; however, there is the possibility of acquiring M2 phenotypes, leading to a tumor-prone microenvironment when exposed to cancer cells^43^. It is worth noting that in every trial regarding the adoptive transfer of macrophages for cancer in our preliminary study, eventually, cancer growth was accelerated. In contrast, ex vivo*-*generated M2 macrophages can be used to promote tissue regeneration; however, there is the risk of their phenotypes reverting to M1 macrophages when they encounter chronic, incurable inflammatory environments. In this regard, macrophage polarization needs to be fully understood with regards to how long the epigenetic mark perpetuates^44^ and how we can potentiate desired phenotypes via ex vivo priming. One way to overcome the uncertainty of phenotype duration is to create genetically ‘fixed’ macrophages. Because the CRISPR system works easily in primary macrophages^45^, the *TNF* gene could be deleted to promote tissue regeneration in concordance with lowering the risk of inducing unnecessary inflammation. The knockout of a critical enzyme or transcription factor may also be useful; for example, *Prkacb* deletion promotes macrophages into M1 phenotypes even when exposed to the cancer environment^46^.

Along with the phenotype duration, the period of substantive activity of transferred macrophages in vivo requires further elucidation. In-depth tracking studies of injected macrophages have yet to be performed. In previous reports using murine iPSC-derived macrophages, the population maintained the macrophage phenotype for at least 1 year^47^. While many immunological studies have shown the persistence of transferred macrophages using valuable mouse models for cell tracing, few studies on therapeutic macrophage transfer have considered the duration of survival with the same function. Preclinical GLP studies should present the distribution data of injected macrophages over time using quantitative PCR following different routes of injections. Intravenously injected macrophages were reported to be trapped in the lungs soon after injection due to entrapment inside the pulmonary capillaries and were rarely observed in tumors; the same result was also observed for MSCs^48–50^. In this regard, intravenously injected macrophages may have beneficial effects even if they are not present in large amounts in the target tissue. These effects are believed to be ascribed to the production of anti-inflammatory cytokines, such as growth factors that ameliorate the damage to organs. However, local injections into the mucosal layer, skin, or a consolidated tissue structure would lead to different biodistributions and pharmacokinetics. For example, gene-corrected macrophages transferred into the lungs of Csf2rb (CD131)-deficient mice were evaluated by transgene-specific PCR tracking^25^. Over a period of 12 months, the proportion of CD131^+^ cells among bronchoalveolar lavage cells increased from 0 to 69% due to the proliferation of the transplanted macrophages. Understanding the in-depth kinetics of injected macrophages along with their mechanistic actions will allow the further use and development of macrophage-based cell therapy.

Another challenge is producing macrophages on a scale that will allow the treatment cost to decrease^3^. Understanding whether and how it will be possible to produce macrophages on a large scale will be an important determinant of whether macrophage cell therapy will transition from a boutique, expensive cottage industry to mass production and take advantage of economies of scale. Manufacturing facilities should provide tightly unified protocols to produce macrophages with stable and consistent phenotypes. At least ~10^9^ M-CSF-derived macrophages should be produced from a patient in one leukapheresis^51–53^, but more importantly, macrophages should be obtained from different sources, including hematopoietic stem cells. Based on the trend that the development of allogeneic therapies has drastically increased in recent years^54^, stable and unified protocols for genetic modification of different sources of macrophages are needed.

### Genetic engineering

As described throughout this review, genetic engineering of macrophages has much therapeutic value. To date, the best way to obtain a specific knockout is mediated by CRISPR/Cas9 complexes. The delivery of ribonucleoprotein into ex vivo-generated macrophages via electroporation is very efficient and safe^45^ and thus will be used to provide therapeutic macrophage sources from allogeneic as well as autologous donors. The advantage of electroporation is that it does not alter the phenotype of macrophages itself. However, it is more complicated in the case of protein overexpression, such as CAR-M. The current successive approach to introduce exogenous DNA sequences is the use of a viral delivery system, which strongly changes macrophages into the M1 phenotype. This is helpful in treating cancer but might be a negative factor for the treatment of regenerative diseases. Further studies are needed to obtain efficient genetic engineering in primary macrophages in the future.

### Future directives

**Fig. 3:**
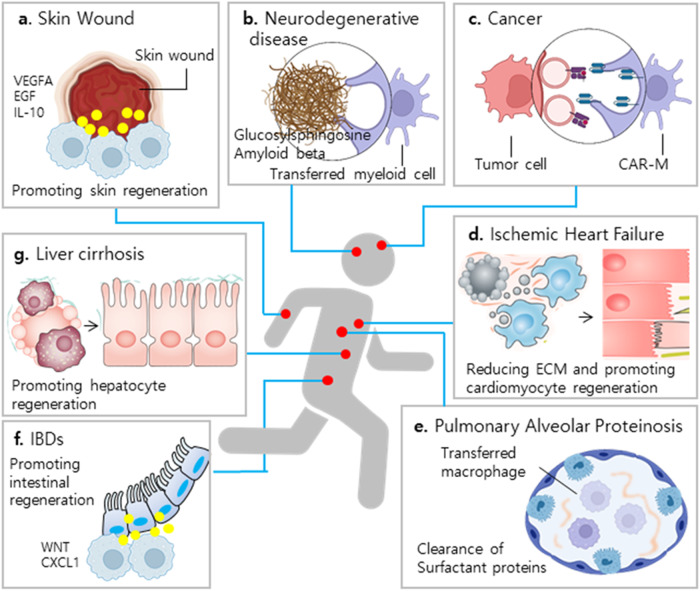
New therapeutic frontiers in macrophage-based cell therapy. The outstanding phagocytic and wound-healing abilities of macrophages will extend the scope of target diseases for macrophage-based cell therapies in the future. **a** Ex vivo activated PBMC-derived macrophages promote skin wound healing via the secretion of growth factors and anti-inflammatory cytokines^21^. **b** Transferred macrophages efficiently clear accumulated neurotoxic materials^30^. **c** Genetically engineered CAR-M eradiate cancer cells^16^. **d** A specific subtype of macrophages reduces ECM contents around the heart injury site and promotes regeneration^23,41^. **e** Transferred macrophages clear excessive surfactant and resolve the pathology of PAP^25^. **f** Macrophages secrete CXCL1 and WNT to coordinate damaged intestinal epithelial cell regeneration^28^. **g** Regenerative macrophages can resolve fibrosis and promote hepatocyte regeneration^24,32^. PBMCs peripheral blood mononuclear cells, ECM extracellular matrix, PAP pulmonary alveolar proteinosis, IBD inflammatory bowel disease, CAR-M chimeric antigen receptor macrophages, VEGFA vascular endothelial growth factor A, EGF epidermal growth factor.

Macrophages have great potential as cellular therapeutic sources (Fig. 3). Their outstanding regenerative capacity could directly aid tissue reconstruction in injured organs, such as the intestines, skin, liver, heart, kidneys, and lungs. Their phagocytic ability could be used to clear cancer cells or neurodegenerative materials as well as infectious agents. As efficient pro-inflammatory regulators, they could be applied to suppress inflammation and deliver active tissue-healing substances. Based on these unique features of macrophages compared to other cell types, more data on safety need to be produced in an effort to make them available for use. In the future, gene editing will be used to obtain purpose-oriented macrophage phenotypes. Although numerous hurdles will need to be addressed, the inherent nature of macrophages will extend their application to new therapeutic frontiers.

### Supplementary information

Supplementary tables
